# Total and differential ionization cross sections in collision between nitrogen atom and singly charged sodium ion

**DOI:** 10.1038/s41598-023-41134-0

**Published:** 2023-08-28

**Authors:** M. Al-Ajaleen, K. Tőkési

**Affiliations:** 1 Institute for Nuclear Research (ATOMKI), Debrecen, 4026 Hungary; 2 Doctoral School of Physics, University of Debrecen, Egyetem tér 1, Debrecen, 4032 Hungary; 3 Centre for Energy Research, Budapest, Hungary

**Keywords:** Physics, Atomic and molecular physics

## Abstract

We present a theoretical study of the ionization of nitrogen atom by a singly charged sodium ion using the classical trajectory Monte Carlo method. Although we suffer from a lack of cross section data of this collision system, the knowledge of the basic cross sections is essential in fusion science, because this reaction has potential applications in the diagnostic of magnetically confined fusion plasmas. In our investigations, the Na^+^–N collision system is reduced to a three-body problem. The interaction between the collision partners is described by the Garvey-type model potential. The results of our study provide insight into the dynamics of singly charged sodium–nitrogen interactions. The total cross sections are presented in the impact energy range between 10 keV and 10 MeV and compared them with the available experimental data. The single and double differential cross sections are presented at 30, 40, 50 and 60 keV energies related to the energies of the plasma diagnostic used in the nuclear fusion.

## Introduction

Ionisation is one of the phenomena that have a huge role in radiation physics and in studying the structure of atoms and molecules^1^. Moreover, it also has significant importance in the study of the fusion plasma. In tokamak magnetically confined fusion plasmas, the excited impurity ions distributions and concentrations have a big impact on the plasma edge profiles. To measure the plasma parameters (i.e., temperature, impurity concentrations and density) as well as plasma turbulence^2^, a diagnostic method which requires injecting a fast neutral atomics beam into the edge plasma is used for high spatiotemporal resolution. Many atoms were used as neutral diagnostic beams such as helium, lithium, and nitrogen^2–6^. Moreover, in tokamak reactors, the process of nitrogen seeding is utilized to cool down the edge plasma^7^. An emission line is generated by the collision of the neutral atomic beam with the constituent particles of the plasma, the emission lines provide a better understanding of the edge plasma profiles. The accurate diagnostic methods rely on the knowledge of the highly accurate cross-sections of the collision systems in the edge plasma.

For the diagnostic purposes, helium was the first choice to be used as a diagnostic beam. Many theoretical models were used to study the ionisation processes in collision systems involving helium. However, comparing the theoretical and experimental data of the double differential cross-section (DDCS) we find a significant discrepancy between them. In studying the DDCS versus the angular distribution of electrons ejected from helium atom, Madison^8^ and Manson et al^9^ used a Hartree–Fock potential to obtain bound and continuum electron wave functions to calculate the differential cross-section in the plane-wave Born approximation for protons and electrons projectiles respectively. Their results had a good agreement with the experimental data at high impact energies. The continuum-distorted-wave-eikonal-initial-state (CDW-EIS) approximation was used by Fainstein et al^10^ to study the differential cross-section in the interaction between helium atom and bare projectiles. The CDW-EIS is a simplification of the CDW method^11^, while the CDW method uses full electronic Coulomb wavefunction, the CDW-EIS method uses the asymptotic behaviour of the mentioned wavefunction (i.e., the logarithmic Coulomb phase)^12^. The results of Fainstein et al^10^ had a slight disagreement with the experimental data at certain ejection angle. The three-body distorted-wave Born approximation (3DWBA) method which was used to study the ionization of helium by low-energy electrons in coplanar geometry was suggested by Jones and Madison^13^. The 3DWBA includes the interaction between the incident electron and the ejected electron, this inclusion led to a high agreement with the experimental data. On the other hand, Jones et al^14^ used the wave functions introducing by Alt and Mukhamedzhanov^15^ and Berakdar^16^ in the 3DWBA, and the calculated results show less agreement with the experimental data.

The study by Wolfrum et al.^17^ was performed to investigate the usage of sodium as diagnostic beam for plasmas as an alternative for lithium beam. They chose sodium over lithium for many advantages of sodium. The main advantages are as follows: lower emitter temperature, larger charge exchange cross section for collision with helium and carbon impurities (see more in ref.^17^). Anyhow, in the recent years, neutral alkali beams (namely Li and Na) with an energy of around 60 keV were used to diagnose the magnetically confined plasmas in the scrape-off layer and the edge, namely, to measure the plasma turbulence and density profile of the plasma electron^18–23^.

In this work, we present the classical treatment of the ionisation of nitrogen (N) atom by a singly charged sodium ion. The ${Na}^{+}-N$ collision system is reduced to a three-body problem using a Garvey-type distance-dependent model potential^24,25^. The bound electrons and the core of the sodium ion (${Na}^{+}$) are treated as a single body. The same procedure is applied to nitrogen atom $N$. The active electron is treated as one body and the core, and the remaining bound electrons of the nitrogen atom are considered as a single body. The calculations were performed using the classical trajectory Monte Carlo (CTMC) method^26^. We present the total cross sections in the impact energy range between 10 keV and − 10 MeV and compared them with the available experimental data. We also present the single and double differential cross sections of ${Na}^{+}-N(2p)$ collision at 30, 40, 50 and 60 keV energies related to the energies of the plasma diagnostic used in the nuclear fusion.

## Results and discussion

In order to study the outer shell ionisation of the nitrogen atom by singly charged sodium ion we performed a large number of classical trajectory simulations. We follow 10^6^ randomly selected individual trajectories for each collision systems.

**Figure 1:**
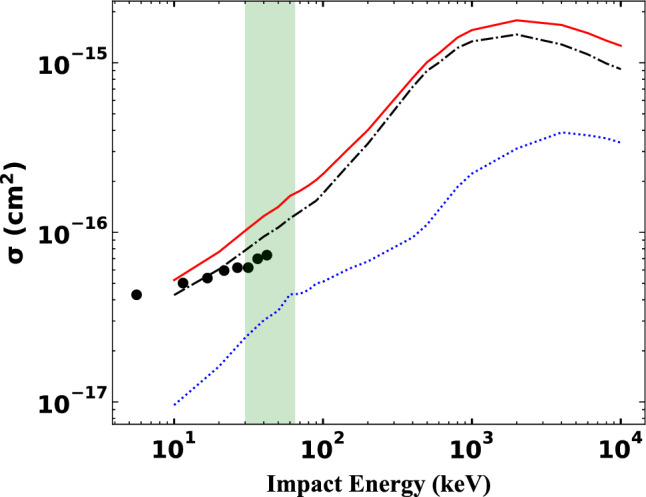
Total ionisation cross section (TCS) as a function of the impact energy in collision between single charged sodium ion with nitrogen atom. Solid red line: present CTMC results of the sum for 1s, 2s and 2p sub-shells; black dashed-dotted line: TCS of N2p sub-shell; blue dotted line: TCS of N2s sub-shell; solid circles: experimental data by Graham et al.^27^.

Figure 1 shows our CTMC results for the total ionisation cross section (TCS) as a function of the impact energy in wide range of projectile impact energies in comparison with, according to our best knowledge, the only experimental data by Graham et al.^27^. We note that Graham et al. used molecular nitrogen target in their study, instead of atomic target what we used. In order to compare our results with the experimental data we need to consider the followings. Firstly, a molecular nitrogen contains two equivalent nitrogen atoms, hence we must multiply our calculated cross sections by two. Secondly, the overall ionisation TCS of the ${Na}^{+}-N$ collision system include all the sub-shells, (i.e., 1s, 2s and 2p), and since we used a single particle approximation, the corresponding ionisation cross sections must be multiplied by the number of electrons in the given shell, i.e. TCSs of 2p sub-shell is multiplied by three, TCSs of 1 s and 2 s sub-shells are multiplied by two. The TCS of 1s sub-shell is not plotted in Fig. 1 because its contribution is negligible small. But Table 1 shows some TCS results of 1s sub-shell at selected impact energies. Taken into these corrections and according to the Fig. 1. we can conclude that our calculated total cross sections are in very good agreement with the available experimental data. Moreover, we present the cross sections in much wider projectile energy range than the experimental data as the benchmark cross sections for the forthcoming measurements or calculations.

**Table 1:** Ionisation total cross section of ${Na}^{+}-N(1s)$.

Energy (keV)	σ_1s_ (cm^2^)	σ_tot_ (cm^2^)	(σ_1s_/σ_tot_) × 100%
50	1.80671E−21	1.412355E−16	1.279215E−03
100	1.11745E−20	2.209473E−16	5.057559E−03
600	4.83966E−20	1.137680E−15	4.253973E−03
2000	4.70016E−19	1.780993E−15	2.639068E−02
10,000	4.17600E−18	1.260183E−15	3.313804E−01

We found that the total ionization cross section maximum is around at 2 MeV impact energy. The green shaded area in Fig. 1 covers the energy range that is in interest in the fusion plasma research regarding to the plasma diagnostics. Therefore, in the followings we focus on this limited energy range to analyse the differential cross sections (DCSs). According to our results in Fig. 1, we will consider only the 2p sub-shell in the differential cross sections results.

**Figure 2:**
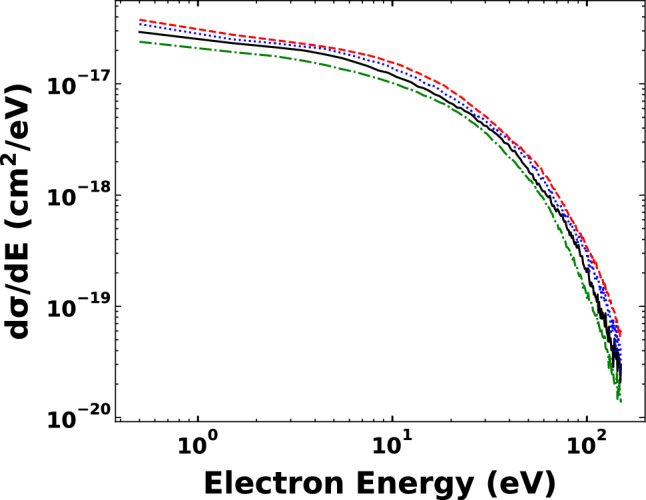
Energy distribution of the electrons emitted from nitrogen in ${Na}^{+}- N(2p)$ collisions as a function of the projectile impact energies. Green dashed-dotted line: 30 keV, black solid line: 40 keV, blue dotted line: 50 keV, red dashed line: 60 keV.

Figure 2 shows the energy distribution of the electrons emitted from nitrogen atom in ${Na}^{+}- N(2p)$ collisions. The single differential cross sections (SDCS) are compared at different projectile impact energies from 30 keV till 60 keV. In all projectile impact energies, the low energy electrons are dominant. The SDCSs are very close to each other and follow the same trend. This is due to the close values of the impact energies under our investigation. We found that around 10 eV, the SDCS starts to decrease rapidly.

**Figure 3:**
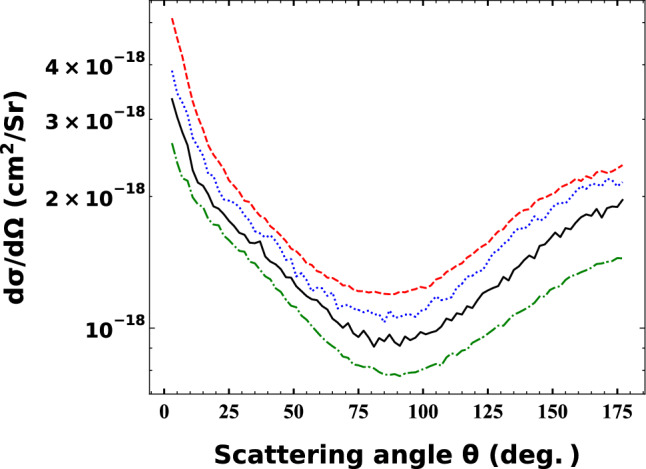
Angular distribution of the electrons emitted from nitrogen atom in ${Na}^{+}- N(2p)$ collisions as a function of the projectile impact energies. Green dashed-dotted line: 30 keV, black solid line: 40 keV, blue dotted line: 50 keV, red dashed line: 60 keV.

Figure 3 shows the angular distribution of the electrons emitted from nitrogen atom in ${Na}^{+}- N(2p)$ collisions as a function of the projectile impact energies. According to the energy dependence of the total ionization cross sections the higher the impact energy the higher the angular differential cross sections. The dominant electron yields can be found in the lower scattering angles. At the same time, we note, that significant electron yield is observable at backwards angles. The cross section minimums are at emission angles range around 90°.

**Figure 4:**
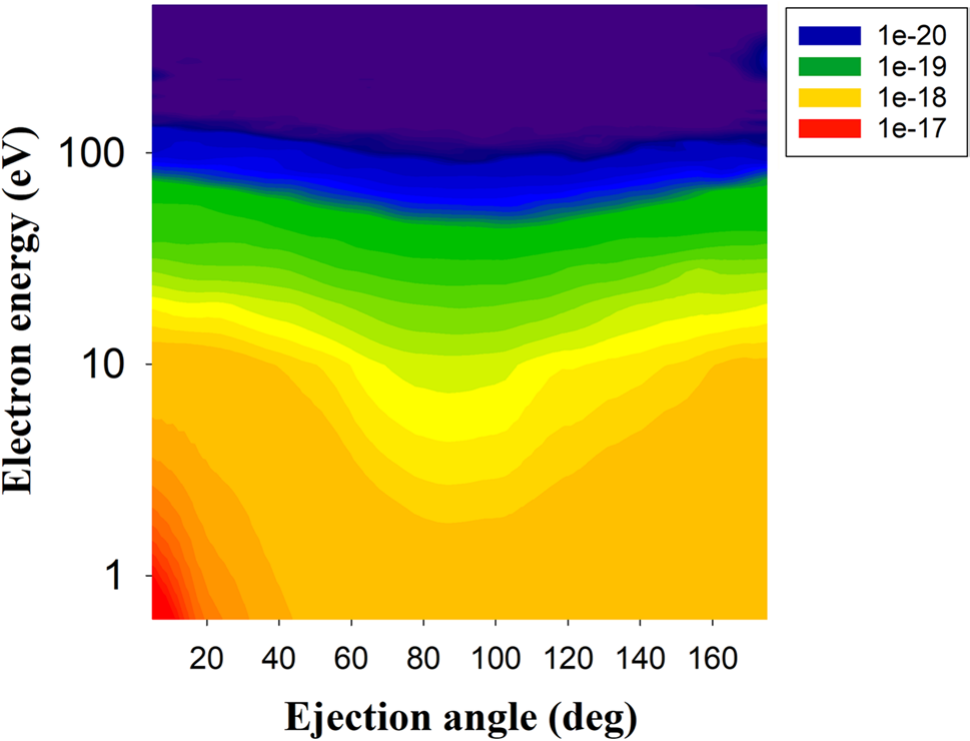
Double differential cross section (DDCS) as a function of the ejected electron energy and scattering angle of the ionised electrons from nitrogen in ${Na}^{+}- N(2p)$ collision system at impact energy 30 keV.

**Figure 5:**
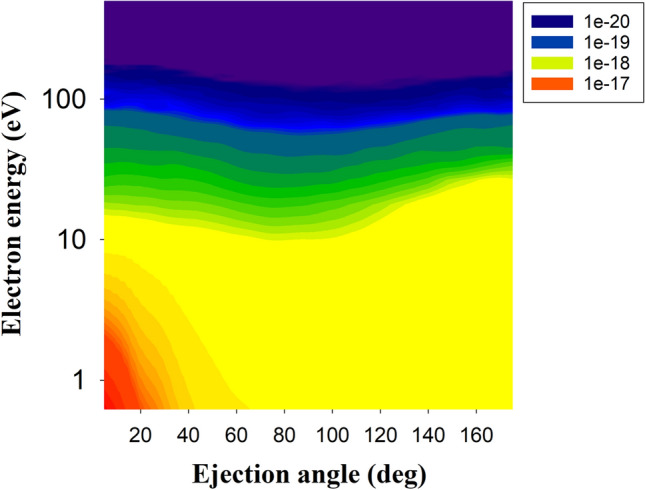
Same as Fig. 4 but for impact energy of 60 keV.

Figures 4 and 5 display the contour plot of the double differential cross section (DDCS) of the ejected electrons from nitrogen in ${Na}^{+}- N(2p)$ collisions at impact energies of 30 keV and 60 keV, respectively. In both cases the highest DDCSs are obtained for electrons with energies less than 10 eV and ejection angles less than 20°. For electrons with energies larger than around 15 eV, the DDCS for both impact energies exhibit almost identical results over all ejection angles. However, a clear difference is observable between the results at 30 keV and 60 keV in the angular range of 60°–120°. Here, the DDCS for the 30 keV impact energy shows lower values compared to the DDCS for the 60 keV impact energy and we found that the DDCS values are almost identical for all scattering angles for 60 keV impact energy.

**Figure 6:**
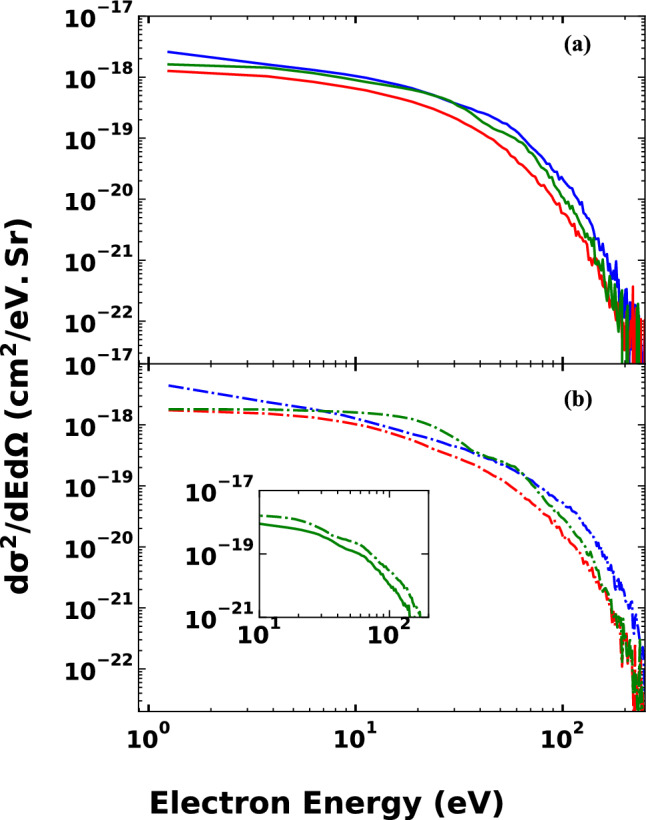
Double differential cross section (DDCS) of the ionised electrons from nitrogen in ${Na}^{+}- N(2p)$ collision as a function of ejected electron energy. (**a**) Impact energy 30 keV, Blue solid line: the scattering angle is 30 ± 15; red solid line: the scattering angle is 90 ± 15°; green solid line: the scattering angle is 150 ± 15°; (**b**) Impact energy 60 keV, blue dotted-dashed line: the scattering angle is 30 ± 15°; red dotted-dashed line: the scattering angle is 90 ± 15°; green dotted-dashed line: the scattering angle is 150 ± 15°.

In Fig. 6, the DDCS is presented as a function of the energy of the ejected electron at impact energies of 30 and 60 keV. The DDCS curves exhibit a complex behaviour with respect to the impact energy and ejection angle. The highest energy DDCS curve, below 5 eV, is attributed to the impact energy of 60 keV and ejection angle of 30 ± 15°. In contrast, the lowest energy DDCS curve over all ejection energies is due to the impact energy of 30 keV and ejection angle of 90 ± 15°.

**Figure 7:**
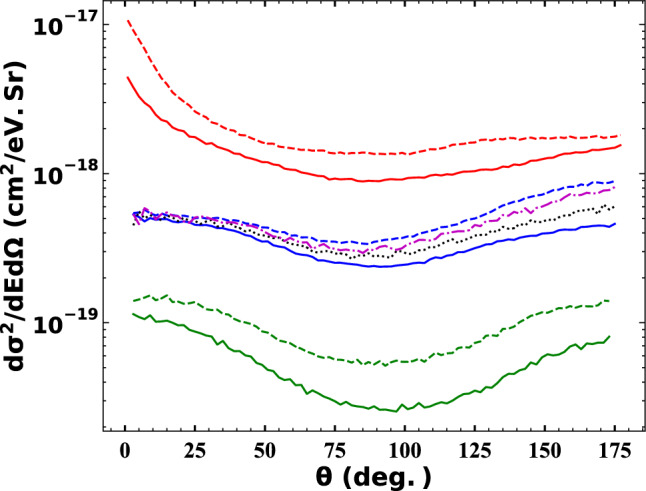
Double differential cross section (DDCS) of the ionised electrons from nitrogen in ${Na}^{+}- N(2p)$ collision system as a function of the scattering angle. Red solid line: impact energy 30 keV, electron energy $0<{E}_{e}\le 12\, \mathrm{eV}$; red dashed line: impact energy 60 keV, electron energy $0<{E}_{e}\le 12\, {\rm eV}$; blue solid line: impact energy 30 keV, electron energy $12<{E}_{e}\le 50\, \mathrm{eV}$; black dotted line: impact energy 40 keV, electron energy $12<{E}_{e}\le 50\, \mathrm{eV}$; magenta dashed-dotted line: impact energy 50 keV, electron energy $12<{E}_{e}\le 50 \,\mathrm{eV}$; blue dashed line: impact energy 60 keV, electron energy $12<{E}_{e}\le 50\, \mathrm{eV}$; green solid line: impact energy 30 keV, electron energy $50<{E}_{e}\le 100\, \mathrm{eV}$; green dashed line: impact energy 60 keV, electron energy $50<{E}_{e}\le 100 \,\mathrm{eV}$;

As a general trend, all DDCS curves start with the highest values at lower energies and decrease slowly until the energy of the electron reaches around 20 eV, except for the energy DDCS curve at 60 keV impact energy and scattering angle of 150 ± 15°, which maintains an almost constant values in this energy range. When the electron energy exceeds the 20 eV, DDCSs decrease drastically. Furthermore, we note that in the energy spectra we clearly see the signature of the so called Fermi shuttle type ionization process (see the inset of the Fig. 7.)^28–31^. Fermi shuttle type ionization occurs when the ejected electron can be able to scatter on both the projectile and target core a few times during the motion of the projectile. In each collision with the projectile core the electron gains energy (accelerated) and after a few back and forward collision can reach a relatively large energy. The signature of the accelerated electrons can be seen in the energy spectra as an enhanced electron yield at the corresponding energies. The idea of the Fermi acceleration goes back to 1949 when Fermi^32^ proposed a hypothetic scheme as a possible origin of the high energy cosmic rays. In this proposed scheme giant electro-magnetic fields, moving against each other in space, can accelerate the charged particles to very high energies in long sequences of reflections. Later it was shown that this type of “ping- pong” game can also observable using a much smaller microscopic fields of atoms, molecules, or clusters^28–31,33–38^, where even a short sequence of scattering events can hold out a very interesting observations.

Figure 7 shows the angular distribution of the ejected target electron in the certain energy range for the Na^+^-N(2p) collision at impact energies of 30, 40, 50, and 60 keV. The DDCSs are the highest for ejected electrons having energies below 12 eV at small scattering angles. With higher scattering angles the DDCS values decrease until they reach 60 degree and above 60 degree the cross sections are almost constant. Moreover, at impact energy of 60 keV, the DDCS values are higher than those obtained at 30 keV for all angles and electron energies.

According to the energy distribution of the ejected electrons, for the ejected electrons with energies in the range of 50–100 eV, the cross section values are the smallest values. The forward and backward scattering cross sections are in the same order. The minimum cross sections are at the scattering angle of 90°.

For ejected electrons with energies in the range of 12–50 eV, an interesting trend is observed. The DDCS for all impact energies are almost equal at low angles, but as the angles increase, the cross sections, for all impact energies, start to diverge from each other. The maximum separation between the curves of the four energies occurs at angles near 180 degrees. For the DDCS obtained at an impact energy of 30 keV, the forward scattering is a bit larger than the backward scattering, whereas for the DDCS obtained at an impact energy of 60 keV, the forward scattering is smaller than the backward scattering. These results suggest that by increasing the impact energy, more backscattered electrons are ionised compared to forward scattering in this energy range.

## Theoretical model

**Figure 8:**
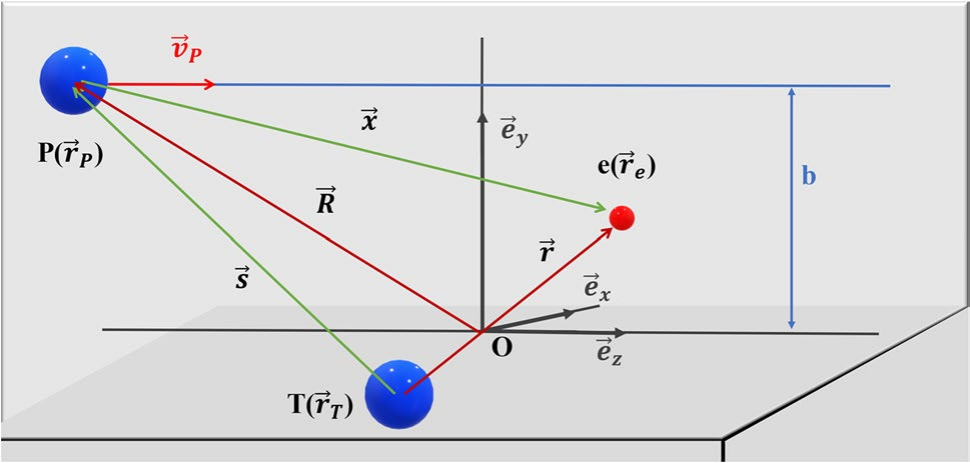
Schematic diagram of the three-body collision system in study. The relative positions vectors are given by $\overrightarrow{r}={\overrightarrow{r}}_{e}-{\overrightarrow{r}}_{T}$, $\overrightarrow{x}={\overrightarrow{r}}_{e}-{\overrightarrow{r}}_{P}$ and $\overrightarrow{s}={\overrightarrow{r}}_{P}-{\overrightarrow{r}}_{T}$. The vector $\overrightarrow{R}$ is the distance between the projectile and the centre of mass of the target system, ${\overrightarrow{v}}_{P}$ is the target velocity and $b$ is the impact parameter.

In this work, the ion-atom collisions are modelled by the classical trajectory Monte Carlo (CTMC) method by sampling randomly the initial conditions of colliding particles and solving the equations of motion numerically. In our investigations, the Na^+^-N collision system is reduced to a three-body problem. The interaction between the collision partners is described by the Garvey-type model potential. The three-body system consists of the projectile, ${Na}^{+} (P)$, the active target electron (e) and the target core (T) including the target nucleus and the remaining inactive target electrons (see Fig. 8.).

The potential energy in interaction between the projectile (P), target core (T) and the electron (e) using the Garvey-type distance-dependent model potential^24,25^ can be written as:

1 $$ \begin{array}{*{20}c} {V\left( {s,x,r} \right) = \frac{{Q_{P} \left( s \right)Q_{T} \left( s \right)}}{s} + \frac{{Q_{T} \left( r \right)q_{e} }}{r} + \frac{{Q_{P} \left( x \right)q_{e} }}{x},} \\ \end{array} $$

where $q_{e}$ is electron charge, $\zeta = s,r,x$ is the distance between the colliding particles, and $Q\left( \zeta \right)$ is the distance dependent nuclear charge given by the following equation:

2 $$ \begin{array}{*{20}c} {Q\left( \zeta \right) = Z - \left( {N - 1} \right)\left( {1 - \Omega \left( \zeta \right)} \right),} \\ \end{array} $$

where Z is the atomic number, N is the total number of electrons in the atom or ion, and the function $\Omega \left( \zeta \right)$ is the screening potential given by,

3 $$ \begin{array}{*{20}c} {\Omega \left( \zeta \right) = \left[ {\left( {\eta /\xi } \right)\left( {e^{\xi \zeta } - 1} \right) + 1} \right]^{ - 1} .} \\ \end{array} $$

The role of the parameters $\eta$ and $\xi$ is to minimize the energy of a given atom or ion. In Table 2, we present the parameters for our system.

**Table 2:** Garvey model potential for nitrogen core and Na^+^.

	N (CORE)	Na^+^
Z	7	11
N	7	11
$\eta$	2.27	2.5031
$\xi$	1.179	1.3197

The parameters $\eta_{ }$ and $\xi$ can be obtained as:

4a $$ \begin{array}{*{20}c} {\eta_{ } = \eta_{ }^{\left( 0 \right)} + \eta_{ }^{\left( 1 \right)} \left( {Z_{ } - N_{ } - a} \right)} \\ \end{array} $$

4b $$ \begin{array}{*{20}c} {\xi_{ } = \xi_{ }^{\left( 0 \right)} + \xi_{ }^{\left( 1 \right)} \left( {Z - N - a} \right)} \\ \end{array} $$

where $a$ equals 1 for projectile and 0 for target, the parameters $\eta_{X}^{\left( 0 \right)} ,\eta_{X}^{\left( 1 \right)} ,\xi_{X}^{\left( 0 \right)} ,\xi_{X}^{\left( 1 \right)}$ are presented by Garvey et al^24^.

**Figure 9:**
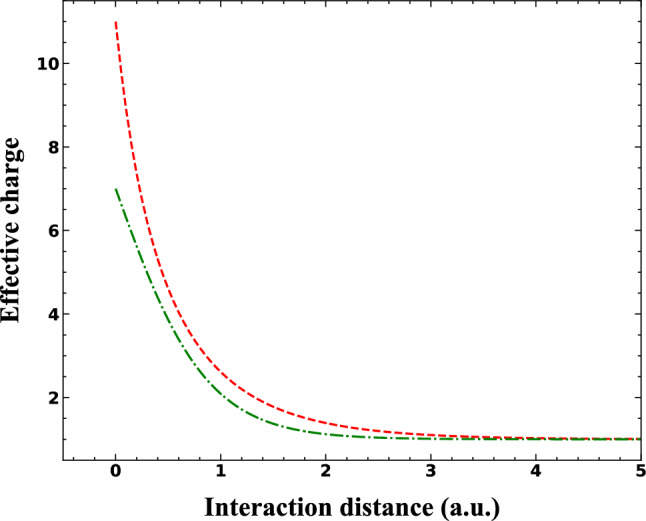
The distance dependent effective charge as a function of interaction distance between the active electron of nitrogen and both the nitrogen core (green dotted-dashed line) and sodium ion (red dashed line).

The screening potential advantage is to consider the distance dependent charge of the projectile and the target core. The effective charge for sodium ion and nitrogen core for both small and large values of $\zeta$ can be seen in Fig. 9.

After obtaining the equations of motion by applying the Hamiltonian to the system, we employed the CTMC method to solve these equations numerically using the adaptive Runge–Kutta method. The step size utilized was dependent on the initial parameters of all particles. For the projectile ion, we defined its initial parameters with respect to the centre-of-mass of the target system. The position was determined by the initial distance ${R}_{0}$ and the impact parameter $b$, which was randomly selected within the interval $\left[0,{b}_{max}\right]$ as illustrated in Fig. 8. The initial momentum was determined by the impact velocity ${v}_{P}$, and the initial direction was chosen to have a z-component exclusively. Concerning the active electron of the target, it was initially confined to the nucleus and was subjected to a non-columbic potential. Reinhold and Falcón^39^ provide a comprehensive explanation of the methodology for determining the initial parameters of this active electron.

The equations of motion are solved for large number of projectiles with random set of initial conditions, the higher number of projectiles results in reducing the statistical error (SE). The total, the energy, the angular differential single differential and the double differential cross-sections can be evaluated using the following expressions:

5 $$ \begin{array}{*{20}c} {\sigma = \frac{{2\pi b_{max} }}{{N_{tot} }}\mathop \sum \limits_{i = 1}^{{N_{t} }} b_{i} ,} \\ \end{array} $$

6 $$ \begin{array}{*{20}c} {\frac{d\sigma }{{dE}} = \frac{{2\pi b_{max} }}{{N_{tot} \Delta E}}\mathop \sum \limits_{i = 1}^{{N_{t} }} b_{i} ,} \\ \end{array} $$

7 $$ \begin{array}{*{20}c} {\frac{d\sigma }{{d{\Omega }}} = \frac{{2\pi b_{max} }}{{N_{tot} \Delta {\Omega }}}\mathop \sum \limits_{i = 1}^{{N_{t} }} b_{i} ,} \\ \end{array} $$

8 $$ \begin{array}{*{20}c} {\frac{{d^{2} \sigma }}{{d{\Omega }dE}} = \frac{{2\pi b_{max} }}{{N_{tot} \Delta {\Omega }\Delta E}}\mathop \sum \limits_{i = 1}^{{N_{t} }} b_{i} ,} \\ \end{array} $$

where $N_{tot}$ is the total number of projectiles with impact parameters less or equal to $b_{max}$, and $N_{t}$ is the number of trajectories with impact parameter $b_{i}$ satisfying the electron ionisation process. The statistical error for a given measurement has the form,

9 $$ \begin{array}{*{20}c} {\Delta \sigma = \sigma \left( {\frac{{N_{tot} - N_{t} }}{{N_{tot} N_{t} }}} \right).} \\ \end{array} $$

## Conclusion

We have presented a theoretical study of the ionization of nitrogen atom by a singly charged sodium ion using a classical treatment of the collision system. Our work is a gap-filling work, as there are either very limited total cross section data are available or no available differential cross section data for this system. In our investigations, the Na^+^-N collision system was reduced to a three-body problem. The interaction between the collision partners was described by the Garvey-type model potential. The total cross sections were presented in the impact energy range between 10 keV and − 10 MeV and compared them with the available experimental data. The single and double differential cross sections are presented at 30, 40, 50 and 60 keV energies related to the energies of the plasma diagnostic used in the nuclear fusion. These impact energies of the differential cross section ensure that Na^+^ projectile can penetrate the plasma to a suitable depth, higher energies will reduce the interaction probability with the plasma components, while lower impact energy means low penetration distance in the plasma. We have shown that the maximum total ionisation cross section occurs at impact energy around 2000 keV. Moreover, we also showed that the majority of electrons are ejected at lower angles and in back scattering, furthermore, most of the ejected electrons have kinetic energy around 20 eV and below. The results provide a valuable tool for understanding the dynamics of ion-atom collisions and their applications in fusion plasma research. The study also highlights the usage of neutral alkali beams in diagnosing magnetically confined plasmas in the scrape-off layer and the edge.

## Data Availability

The datasets generated during and/or analyzed during the current study are available from the corresponding authors on reasonable request.
